# Undocumented migrants’ access to healthcare in Sweden, and the impact of Act 2013:407

**DOI:** 10.1177/09697330231215947

**Published:** 2023-11-24

**Authors:** Anna O’Sullivan

**Affiliations:** 7643 Marie Cederschiöld University

**Keywords:** Access, healthcare, migrant, nursing ethics, Sweden, undocumented migrant

## Abstract

**Background:**

Research shows that undocumented migrants have difficulties in accessing healthcare. Act 2013:407 came into force in 2013 and entitled undocumented migrants to healthcare that cannot be deferred. To date, studies about undocumented migrants’ access to care in Sweden and the impact of Act 2013:407 are sparse. Hence, the aim of this study was to describe professionals’ experiences of access to healthcare for undocumented migrants in Sweden and the impact of Act 2013:407.

**Methods:**

A qualitative design with semi-structured interviews was employed. Nine interviews were carried out in 2015 with nurses at two NGO healthcare centres for undocumented migrants – and an additional seven interviews in 2022 with staff at an NGO healthcare centre for undocumented migrants and personnel at a regional health and medical care administration. Interpretive description was used for the analyses.

**Ethical considerations:**

Permission to carry out the study was obtained from managers at the participating NGOs and the regional health and medical care administration. Participants received verbal and written information about the study, and informed consent was obtained from all participants.

**Findings:**

Six categories emerged from the analysis: Changes since the Act was introduced, General problems with healthcare access, Care for undocumented migrants – politics and social economy, Lack of knowledge, ‘Healthcare that cannot be deferred’ and Being an undocumented migrant.

**Conclusion:**

Undocumented migrants’ social needs are as great as their needs for healthcare. Healthcare staff are burdened with healthcare cost considerations which affect their judgement of care provision and prioritization. Healthcare staff attitudes towards undocumented migrants affect their access to healthcare. Undocumented migrants in need of healthcare are especially vulnerable due to their legal status, being ill and the fear of being reported and deported. To assure undocumented migrants’ access to healthcare and maintain healthcare ethics, the only possible solution is to provide healthcare based on needs.

## Introduction

Healthcare for undocumented migrants in Sweden is a debated issue – in the media, among healthcare professionals, healthcare associations and by non-governmental organizations (NGOs) working with human rights. Act 2013:407 came into force in 2013 and entitled undocumented migrants to healthcare that cannot be deferred. Before Act 2013:407 undocumented adult migrants had no legal rights to subsidized healthcare but could access emergency care at full price; hence, Act 2013:407 expands their access to subsidized healthcare. Research shows that undocumented migrants have difficulties in accessing healthcare. To date, studies about undocumented migrants’ access to care in Sweden and the impact of Act 2013:407 are sparse. Hence, the current study describes professionals’ experiences of access to healthcare for undocumented migrants in Sweden and the impact of Act 2013:407. A qualitative design with semi-structured interviews was employed for the study.

## Background

In 2021, around 3.3 million people immigrated to Europe, approximately two-thirds from non-European countries.^1^ In 2015, the number of people who applied for asylum in Sweden was the highest ever, with over 160,000 people. Political decisions have since affected the possibility of applying for asylum, and the number of asylum seekers has decreased. A new law was introduced in 2016 that restricted permanent residency and family reunion for asylum seekers (SFS 2016:752),^2^ justified by political leaders to ease the increased pressure on authorities and social welfare services. The act was introduced due to the increase of refugees to Sweden in 2015, labelled the ‘refugee crisis’ – regarded as a crisis for the receiving country, rather than a crisis for the people seeking asylum (SOU 2017:12).^3^ Restrictions during the pandemic caused this number to decrease further in 2020 and 2021, and in 2021 it was the lowest in the entire 2000s – just over 11,000 people, a quarter of the applications were deemed to have grounds for asylum.^4^

In Sweden, an undocumented migrant is defined as a person staying in Sweden without a residence permit. The person may have applied for asylum or a residence permit but had their application rejected and chosen to remain in Sweden anyway. It could also be that a tourist visa expired, or they may be victims of trafficking – or have never applied for asylum or a residence permit at all. Although no one really knows how many undocumented migrants there are in Sweden today, in 2010, the National Board of Health and Welfare estimated that there are between 10,000 and 35,000 people.^5,6^

Undocumented migrants can be considered as a particularly vulnerable group, due to not having basic human needs fulfilled, such as an acceptable living standard with regard to housing, nutrition and security^7,8^ or possible traumatic experiences of war, violence and forced migration.^9^ Migrants are said to initially have better health than the average population in their country of birth and the host country, but studies show that migrants’ health advantage diminishes over time and their health status becomes equal to or worse than the native population’s; this is referred to as the ‘healthy migrant effect’.^10^ The healthy migrant effect is not seen in refugees due to their experiences from their home country, refuge and conditions in refugee camps.^11^ Studies have shown health issues with communicable diseases, including respiratory and gastrointestinal infections and infectious diseases such as HIV and TBC,^12^ as well as non-communicable diseases, including chronic conditions.^13^ Mental health issues have been shown to be especially prevalent.^7,14^ There is a high prevalence of PTSD in undocumented migrants and the same is true of other psychiatric conditions such as anxiety and depression.^8,9,14^ Furthermore, the living situation as an undocumented migrant – with the constant threat of being reported and deported back to their home country to an uncertain future — is a further source of stress.^7,8,14^

Swedish Act 2013:407,^15^ focusing on health and medical care for certain foreigners living in Sweden without necessary permits, came into force in 2013. The Act specifies that persons aged 18 years or older living in Sweden without necessary permits, that is, undocumented adult migrants, have a right to subsidized ‘care that cannot be deferred’. Before Act 2013:407, undocumented adult migrants had no legal rights to subsidized healthcare but could access emergency care at full price; hence, Act 2013:407 expands their access to subsidized healthcare. Prior the act undocumented migrants depended on assistance with healthcare from non-governmental organizations (NGOs), either by the NGO intermediation of regular care or care provided by volunteers in the NGO network. Since 2008, this right to healthcare has been extended to include asylum-seekers. ‘Care that cannot be deferred’ includes certain health- and dental care, maternal healthcare, contraceptive advice, abortion care and a health examination. In addition, the county councils are able to offer care up to the same level as for residents, although this is a possibility and not a requirement.^5,15^ The Swedish National Board of Health and Welfare (NBHW) published a report^16^ about the concept ‘care that cannot be deferred’ in 2014 and found that it is not ethically and medically possible or appropriate to indicate which diagnoses, conditions or measures are covered by the concept. The investigation also determined that the concept ‘care that cannot be deferred’ is not compatible with healthcare professionals’ ethics, is not medically applicable in healthcare and risks endangering patient safety. What constitutes ‘care that cannot be deferred’ must be decided for each individual case by the attending physician or dentist.^16^

Previous studies have reported undocumented migrants’ restricted access to healthcare. A range of individual, institutional and legal barriers to access healthcare for undocumented migrants have been described.^7,17–20^ Act 2013:407 entitles undocumented migrants to certain subsidized healthcare; however, it is unknown what access to healthcare undocumented migrants in Sweden actually have. It has been nearly a decade since Act 2013:407 came into force and little is known about its impact. Therefore, the aim of this study was to describe professionals’ experiences of access to healthcare for undocumented migrants in Sweden and the impact of Act 2013:407, which legally entitles undocumented migrants to care that cannot be deferred.

## Methods

A qualitative research method with semi-structured interviews was employed. Interpretive description^21^ was used for the analysis, which is a viable approach for generating knowledge applicable to clinical practice.

## Participants and data collection

In 2015, a purposeful sample of nine nurses who were working – or had worked – at one of two NGOs (non-governmental organizations) healthcare centres for undocumented migrants was selected to partake in a study for a master’s thesis in nursing sciences, which was never published. The sample included eight women and one man, aged 31–65 years, with 8–40 years professional nursing experience and 8 months–16 years experience working in care for undocumented migrants. In 2022, a complementary purposeful sample of seven participants was made to complement the previous sample from 2015. The sample consisted of nurses, a dental nurse and a counsellor working at an NGO healthcare centre for undocumented migrants or at a regional health and medical care administration. The participants were six women and one man, aged between 38 and 64 years, with 11–38 years professional experience and 1, 5–14 years of experience in care for undocumented migrants. Participants from the NGO healthcare centres had regular contact with undocumented migrants, healthcare staff and healthcare providers regarding care for undocumented migrants. The participants from the regional health and medical care administration mainly had regular contact with healthcare providers and healthcare staff, with a focus on asylum seekers as well as undocumented migrants.

Data was collected through semi-structured individual interviews following an interview guide. In 2015, six interviews were face-to-face, and three by phone, while in 2022, all interviews were via video call using Microsoft Teams. The interviews were on average 30 min long, with the material in its entirety consisting of 141 pages of text. The interviews were recorded, transcribed verbatim and then read through to verify consistency with the recordings.

## Data analysis

Interpretive description was used for data analysis, due to its specificity to nursing epistemology. In interpretive description is the rich and complex nature of the human experience acknowledged, and both shared and diverse individual experiences are valued.^21,22^ Initially, the material was read to obtain an overall picture, then it was read again to discover patterns and deepen the understanding. Thereafter, the text was broadly coded, identifying meanings and variations of contextual descriptions of undocumented migrants’ access to health care and the impact of law 2013:407, which were organized into patterns of experiences. These were interpreted through a process of asking questions such as ‘what is the underlying meaning of this or what can we learn from this?’, in accordance with interpretive description.^21^ Finally, all raw data were read again to look for contradictions to the six themes that had emerged from the analysis.

## Ethical considerations

Permission to carry out the study was obtained from managers at the NGO healthcare centres for undocumented migrants and the regional health and medical care administration. The participants received both verbal and written information about the study, and informed consent was obtained from all participants. They were informed that they could, without giving a reason, withdraw their participation at any time. In Swedish practice, ethical permission for research is applied for in accordance with Swedish law (Act (2003:460) on ethical review of research involving humans), if you intend to handle sensitive personal data or the research involves a physical intervention or aims to influence or poses an obvious risk of harming the research subject physically or psychologically. Ethical approval was not deemed required since no risk of harming participants could be foreseen, and the research did not involve any sensitive personal data. The participants in this study are professionals, and the research question concerned the professionals’ work experiences. The recorded interviews were coded so participants could not be identified. All data were presented on a group level, and citations were de-identified to maintain participant anonymity.

## Findings

The analysis resulted in six categories: Changes since the Act was introduced, General problems with healthcare access, Care for undocumented migrants – politics and social economy, Lack of knowledge, ‘Healthcare that cannot be deferred’ and Being an undocumented migrant.

## Changes since the act was introduced

A clear improvement in undocumented migrants’ access to healthcare has been described connected to the introduction of Act 2013:407. It has become easier to argue for undocumented migrants’ lawful right to healthcare – and healthcare staff have greater knowledge about undocumented migrants’ rights than before the act. The act also raised awareness for other groups that were not granted access to healthcare by the act, for example, European migrants, migrants on limited work permits and relatives (of residents who have immigrated to Sweden with temporary residential permits). The participants in the latter interviews raised a concern regarding a changed climate in society, towards more restrained immigration politics and a less accepting climate in society with regard to immigration, and especially to undocumented migrants. However, participants described access to healthcare for undocumented migrants as still being problematic and reported that, although the act had had some impact, they continued to see the same problems related to access to healthcare as they had seen before the act, described in the findings below.

## General problems with healthcare access

The participants described difficulties in the healthcare of undocumented migrants, such as communication difficulties or misunderstandings, which are universal for all patients and not unique to undocumented migrants nor influenced by Act 2013:407. They expressed difficulties related to contact regarding healthcare for the undocumented migrants due to language barriers, for example, they could not book an appointment by phone because they did not understand what the person on the other end of the phone was saying.

Furthermore, the participants reported that certain care was not available due to a lack of available appointments, for example, in dental care and psychiatric care. Participants described how access to dental care and psychiatric care could be difficult for all patients due to regional differences and availability, as described by one of the participants:

‘It is the psychiatric patients that you despair about, when they go into the emergency room and are admitted to the ward for one or two days and then discharged without follow-up, it is only to prevent suicide at the time, but they get no treatment since there are no appointments’.

The participants described how undocumented migrants can only get emergency appointments for dental care due to the lack of appointments. The problem was partly specific for undocumented migrants, but asylum seekers were also perceived as being exposed to the problem.

## Care for undocumented migrants – politics and social economy

The participants expressed how undocumented migrants’ access to healthcare is used for migration policy purposes to limit immigration. They described how the main focus in the political discussion about undocumented migrants’ legal right to healthcare is not healthcare itself but rather the question of their legal status and opportunities to stay in the country – with political views against undocumented migrants’ legal right to subsidized healthcare, since this further enables them to stay in the country. This was also discussed by the participants in the context of health and access to healthcare as a human right:

‘The right to healthcare is something that applies to all people, regardless of who they are, and healthcare is used as a migration policy tool to try to control flows in and out of the country by limiting the opportunities for health’.

Participants described how the legislation that exists today puts healthcare staff in a difficult situation; as the Act itself is discriminatory, the healthcare staff are also forced to discriminate. Act 2013:407 does not grant the right to equal healthcare for all residents but limits access to healthcare for undocumented migrants to only include care that cannot be deferred. The level of care they receive depends on legal status and not care need, which also puts pressure on healthcare staff to provide care according to status rather than patient need as stated in their ethical code; one participant summarized it as follows:

‘Care for the undocumented has become a migration problem. It is not a healthcare problem you are talking about; it is about immigration, legal status is what you decide on first and not the needs of the individual’.

Furthermore, the participants described their experiences of how healthcare staff seem to feel a responsibility for the employer’s and society’s finances. They recounted how the finances of healthcare have become the responsibility of healthcare staff, with employers regularly raising the issue of costs and finances. This financial burden on healthcare staff resulted in restricted access to healthcare for undocumented migrants:

‘The economy of healthcare comes before the individual person with needs. It is a cost for Sweden and the taxpayers in Sweden’.

The participants also reported, based on their experience, healthcare staff feel that they are personally responsible for non-payment of healthcare. This is the case for administrative staff and cash register staff in particular, since some of their main duties include registration of visits and invoicing. The participants also felt that healthcare staff seemed to fear making mistakes and being held accountable, which resulted in healthcare staff denying undocumented migrants healthcare when unsure whether they were entitled to subsidized healthcare or if they lacked the ability to pay. One participant described how this impacted undocumented migrants’ access to healthcare and their human right to achieve health:

‘You are afraid of making a mistake, of not behaving ethically towards your own practice and start admitting patients for whom you may not be paid and that comes before behaving ethically towards the patient and looking at the care needs’.

Furthermore, participants described how they experienced hearing healthcare staff’s personal opinions about how undocumented migrants ‘should not be here’ and how they ‘cannot just come here and receive care’ – attitudes described by the participants as based on racism. Participants also reported experiences of how healthcare staff and healthcare providers give the impression that they think you should ‘earn’ the right to healthcare by contributing to society, for example, by working and paying taxes, or by being vulnerable enough to deserve healthcare due to having fled war or by looking poor:

‘There is an attitude among some that you have to earn the right to healthcare as an undocumented person, that not all undocumented migrants are undocumented enough to deserve your pity and for you to be generous with healthcare’.

## Lack of knowledge

The participants described how there is a knowledge gap among healthcare staff about Act 2013:407 and also about the undocumented migrant patient group. They saw this as an obstacle to access healthcare for the undocumented migrants. The participants reported that, as a result of this lack of knowledge, in unclear situations, healthcare staff would rather not provide care than to provide care:

‘With some people, it happens that, based on ignorance or a wrong idea of ​​what an undocumented person is, you let the uncertainty dominate and do not provide care, rather than if you are unsure, you provide care in all cases’.

According to the participants, healthcare staff do not know what it means to be an undocumented migrant, or who is an undocumented migrant and who is not (e.g. a tourist), and there is also a lack of knowledge about what care they are entitled to. The participants explained this lack of knowledge as being a consequence of the relatively small group of undocumented migrants who seek care, so some healthcare providers have never encountered undocumented migrants and therefore lack experience. The participants reported that healthcare staff were restrictive with care when unsure of the patient’s legal status and right to receive care, from fear of making mistakes that they would be held accountable for.

Participants also describe how undocumented migrants’ access to healthcare is hindered by their own lack of knowledge of their healthcare rights. Some undocumented migrants learned of their right when they were asylum seekers or if they went for a free health check-up. Besides this, knowledge is limited and there are no clear ways to share the information with the group, except word of mouth:

‘They come to us because they don’t know their own rights, or they have been in contact with healthcare but have not received the help they feel they need’.

## ‘Healthcare that cannot be deferred’

The participants describe how the concept ‘healthcare that cannot be deferred’ causes many difficult situations and ethical dilemmas for healthcare staff who have to decide what is included in this concept. It becomes difficult, especially if the patient’s care needs are not perceived to be covered, which then affects the patient’s health. One participant described it as follows:

‘You know that there is a limitation in what care undocumented migrants can receive, so there is a risk that you become very focused on that limitation, instead of thinking here is a person with a medical problem, how do I solve this medical problem? Is this person’s medical problem something that we should help with or should we say no?’

The participants gave examples from psychiatric- and dental care, describing how undocumented migrants only received emergency help but no follow-up or other treatment. This was partly explained not only by how psychiatry and dental care resources are stretched but also by the fact that healthcare staff are constrained by the concept of ‘care that cannot be deferred’. The participants described how the concept is sometimes interpreted by healthcare staff as equivalent to emergency care, which – for example, in psychiatry and dental care – leads to undocumented migrants only receiving help when the need for care has become urgent.

According to the participants, the individual treating physician should decide if the patient’s care needs are included under the concept of ‘care that cannot be deferred’. However, a difficulty described by the participants is when the perception of what care is included differs, for example, when the participants consider it care that cannot be deferred, but the physician refuses to give care indicating that it is not included in the concept:

‘We had an undocumented migrant who had been refused a follow-up after a heart attack at a medical clinic, because they believed that it was not included. Care that cannot be deferred is not at all about only emergency conditions, but also, like in this case when a person is fragile after a heart attack, a follow-up is needed and that is something that cannot be deferred’.

The scope of the concept of care that cannot be deferred is described as difficult, and the participants reported a lack of follow-up care, such as rehabilitation, but above all care provided by municipalities such as home care or nursing home care. Municipal care is not covered by Act 2013:407, and it is unclear if subsidized municipal care can be provided. Hence, this type of care is difficult for undocumented migrants to access, as illustrated by one participant’s example:

‘There was one undocumented migrant, a woman in need of daily support from home healthcare, but this woman was sent home from the hospital to a garage where she lives without any further care’.

## Being an undocumented migrant

The participants raised issues with the legal status of undocumented migrants, relating to how the discriminatory legislation of Act 2013:407 obliges healthcare staff to investigate patients’ status to make sure that the person has the right to subsidized healthcare. Participants describe how undocumented migrants are especially vulnerable given their social status, while at the same time being ill and in need of healthcare. Undocumented migrants do not usually want to flag their status and it is difficult to prove that you are undocumented. The participants described how they have witnessed healthcare staff requiring undocumented migrants to identify themselves and if the person had a passport, healthcare staff claimed that the person cannot be an undocumented migrant since they have a passport. Furthermore, the participants described how healthcare staff believe a person is only an undocumented migrant if they have fled from countries in crises, such as war.

Participants highlight undocumented migrants’ fear which prevents them from seeking and accessing healthcare. This fear is related to lack of trust in authorities and concern about being reported and deported to their birth country. In contact with healthcare, their fear causes misunderstandings. Furthermore, participants described how undocumented migrants cannot make demands or be assertive, due to lack of language or knowledge, and how they avoid conflict:

‘You are so scared you do not dare to go, you might need someone to come with you…when information is lacking, just as it is sometimes for all patients who seek medical care, but the vulnerable situation you find yourself in, as an undocumented migrant and not knowing what to expect, is an anxiety-filled situation’.

Participants described how undocumented migrants’ financial circumstances hinder care and how they cannot afford medicines or a healthcare appointment. Social issues were described as dominating, especially regarding children and older people with multiple diseases, and lack of support from municipalities – financially as well as with healthcare and childcare. Furthermore, participants described how children grow up undocumented in Sweden today, and that a parallel shadow society is developed where children live their whole lives undocumented – they go to school, the family do cash-in-hand jobs and they are an integrated part of society, but not fully. Participants further described how older undocumented migrants with serious medical conditions, who have their asylum application rejected and are too ill for deportation, do not receive the care needed. They are often cared for by family members, who may or may not be capable of helping with their care.

## Discussion

The study findings consist of six themes: Changes since the Act was introduced, General problems with healthcare access, Care for undocumented migrants–- politics and social economy, Lack of knowledge, ‘Healthcare that cannot be deferred’ and Being an undocumented migrant.

The findings of general problems regarding healthcare access reflect problems that are common for people with a foreign background and people born in Sweden, for example, communication problems including misunderstandings or not being treated in a desirable way, due to lack of correct information for self-determination. This can be related to the care structure, administrative problems due to language barriers or availability of an interpreter, which influence patients’ understanding of how the care works.^23^

In this study, participants described the political discussion of healthcare access as an incitement for migration. Winter et al.’s^8^ study of undocumented migrants’ access to healthcare showed an underutilization of healthcare services by undocumented migrants and that care was often inadequate. Berlinger and Raghavan^24^ also describe undocumented migrants seeking healthcare to a lesser extent than the rest of the population, since they are younger and unable to seek care, for example, because they cannot take time off work, but mainly due to deportation fears.

This study’s findings showed that healthcare staff are burdened with financial considerations regarding healthcare and that this hinders undocumented migrants’ access to healthcare and care according to needs. This conflict arises due to healthcare staff’s loyalty and sense of duty towards society and its taxpayers, and their employer, versus the patient’s need for and access to healthcare. This also makes it impossible for healthcare staff to work in accordance with their ethical codes of conduct and care ethics. Benjamin and Curtis^25^ discuss the cost of and access to healthcare, describing how the biggest ethical issue in nursing today is that of access to and lack of resources for care – an issue that cannot be solved by individuals or organizations; rather, the way in which welfare, including healthcare, should be used and financed must be resolved at the societal level. Molina-Mula and Pedro-Gomez^26^ raise the injustice of financial considerations opposed to the fundamental right to health and highlight how certain groups are excluded from healthcare based on finance. These authors argue that limiting access to care affects nurses, who cannot ignore their ethical guidelines, responsibilities towards the patient and obligations to meet the population’s care needs, especially the most vulnerable groups. Berlinger and Raghavan^24^ describe healthcare staff’s role as advocates for the patients and their rights to care – a role which gives healthcare staff satisfaction. Furthermore, these authors argue that care for undocumented migrants should be discussed within healthcare centring on ethical dilemmas and difficulties for healthcare professionals. This study’s result of the financial burden put on healthcare staff related to the cost of undocumented migrants access to subsidized healthcare could be seen as reflective of broader trends influenced by neoliberal politics and marketization, which emphasize economic efficiency and cost reduction through competition, and has gradually increased in the Swedish welfare society since the economic crises in the 1990s.^27,28^ This requires that healthcare staff are more critical and empowered to act according to ethics for care, rather than economic considerations and political incitements.

The present study’s findings show that undocumented migrants’ life situation and fear hinder access to healthcare, which is in line with previous studies.^7,17,29^ Larchancé^17^ describes how undocumented migrants’ fear of authorities and healthcare staff’s suspicion towards them constitute difficult obstacles to care for undocumented migrants. Further findings show the difficulty of undocumented migrants’ civil status, which is hard to prove and also difficult to understand without knowledge of undocumented migrants. Furthermore, in the present study healthcare staff were obliged to investigate patients’ status to make sure that the person had the right to subsidized healthcare, and thus act as migration police. This is confirmed by Campbell et al.^29^ who question whether it is ethical for healthcare staff to act as migration police and assess patients’ status to decide whether they should receive care. Presently, there is an ongoing political discussion in Sweden about a proposal that enforces a legal obligation for professionals in the public sector – employees of municipalities or authorities (e.g. healthcare staff, teachers, social workers and librarians) to report people who are staying in Sweden without permits. The proposal states that municipalities and authorities must inform the Swedish Migration Agency and the Swedish Police Agency when they come into contact with persons staying in Sweden without a permit. This would further complicate healthcare staff’s possibilities to work in accordance with ethical codes for care and maintain patient confidentiality.

In line with previous research,^30^ the present study’s findings showed that healthcare staff lack knowledge of undocumented migrants’ rights to healthcare, which hinders access to healthcare. Furthermore, present study findings showed lack of knowledge about the legal right to healthcare among undocumented migrants, which is in line with previous studies.^8,9^ Studies indicate that undocumented migrants’ access to healthcare is influenced by healthcare staff’s personal attitudes and perceptions,^7,17,31^ which is in accordance with the results of the present study. Whether undocumented migrants contribute to society through work and paying taxes influences healthcare staff’s attitudes towards whether undocumented migrants ‘deserve’ the right to healthcare, which influences access to care.^17,31^

## Limitations

The sample of nurses and other healthcare professionals working at NGO healthcare centres for undocumented migrants or at a medical health and care administration, who all had experience with undocumented migrants’ access to healthcare since Act 2013:407 came into force, was considered suitable to address the purpose of the study. The size of the sample was determined by the fact that all those invited chose to participate in the study. Interview one functioned as a test interview but yielded such good results that it was included. The first sample was complemented with seven participants to assure the relevance of previous data for the situation today. The quantity and quality of data were considered sufficient for the study purpose.

The authors’ pre-understanding from previous work with undocumented migrants’ healthcare was considered throughout the study, to limit influencing interpretation of the material. Furthermore, during the analysis work, there was a dialogue between the author and a selection of the participants, to secure the interpretation of data. Additionally, participants’ quotations were used to confirm the interpretations.

The findings of this study are based on professionals’ experiences of undocumented migrants’ access to healthcare in Sweden through their work at NGOs or care administration offices and may be transferable to other care contexts in Sweden; however, transferability to other countries will depend on national acts and regulations. Firsthand experiences of access to healthcare from professionals working in Swedish healthcare or from undocumented migrants would of course have been preferable, but given the precarious nature of being an undocumented migrant and the question of the impact of Act 2013:407, it was deemed viable to include professionals at NGO healthcare centres for undocumented migrants for the study instead.

## Conclusion and implications

Act 2013:407 provides undocumented migrants certain rights to care, but it also legitimates discrimination. The act does not grant equal access to healthcare and demands discrimination depending on legal status. This study shows that healthcare staff are burdened with concerns about the cost of care, which affects their work and assessments of care provision. Healthcare staff’s personal attitudes towards undocumented migrants may affect undocumented migrants’ access to care. The lack of inclusion of municipal care such as home care and nursing home care in Act 2013:407 is noteworthy and excludes undocumented migrants’ access to this type of care.

Social needs of undocumented migrants are as great as their care needs, and help with social needs is limited for undocumented migrants. Living as an undocumented migrant is stressful, with constant fear of being reported and deported, resulting in refraining from seeking care. Even if they do seek care, they may be required to prove their status as an undocumented migrant in order to receive care. Raising knowledge among healthcare staff about Act 2013:407 could perhaps increase understanding of what it means to be an undocumented migrant and thus contribute to greater access to healthcare. The concept ‘healthcare that cannot be deferred’ needs clarification, and a more nuanced discussion about this concept related to patients’ actual care needs, consequences of postponed care and ethical considerations for healthcare staff is required. There is a need to shift focus away from the individual healthcare professional’s responsibility, to a political and societal level, and to a wider discussion about the influence of neoliberalism on the Swedish welfare society. Healthcare professionals, with support from civil society, should continue their work with shedding light on the ethical dilemmas and difficulties for healthcare professionals with regard to Act 2013:407 and diligently work for a change through advocacy. Healthcare professionals should provide care primarily in accordance with healthcare ethics and professionals’ codes of conduct. To uphold care ethics, the only possible solution is to provide care according to needs. The legal framework for care of undocumented migrants today causes ethical dilemmas for healthcare staff in the provision of care for undocumented migrants, who are discriminated against, and their unique human value is not considered.
